# Specifically Expressed Genes of the Nematode *Bursaphelenchus Xylophilus* Involved with Early Interactions with Pine Trees

**DOI:** 10.1371/journal.pone.0078063

**Published:** 2013-10-14

**Authors:** Xiuwen Qiu, Xiaoqin Wu, Lin Huang, Minqi Tian, Jianren Ye

**Affiliations:** 1 Institute of Forest Protection, College of Forest Resources and Environment, Nanjing Forestry University, Nanjing, Jiangsu, China; 2 Jiangsu Key Laboratory for Prevention and Management of Invasive Species, Nanjing, Jiangsu, China; Auburn University, United States of America

## Abstract

As the causal agent of pine wilt disease (PWD), the pine wood nematode (PWN), *Bursaphelenchus xylophilus*, causes huge economic losses by devastating pine forests worldwide. However, the pathogenesis-related genes of *B. xylophilus* are not well characterized. Thus, DNA microarrays were used to investigate differential gene expression in PWN where *Pinus thunbergii* was inoculated with nematodes, compared with those cultured on *Botrytis cinerea*. The microarrays comprised 31121 probes, 1310 (4.2%) of which were differentially regulated (changes of >2-fold, *P* < 0.01) in the two growth conditions. Of these 1310 genes, 633 genes were upregulated, whereas 677 genes were downregulated. Gene Ontology (GO) categories were assigned to the classes Cellular Component, Molecular Function, and Biological Process. The comparative gene expression analysis showed that a large number of the pathogenesis-related genes of *B. xylophilus*, such as pectate lyase genes, cytochrome P450s, UGTs, and ABC transporter genes, were highly expressed when *B. xylophilus* infected *P. thunbergii*. Annotation analysis indicated that these genes contributed to cell wall degradation, detoxification, and the reproduction process. The microarray results were validated using quantitative RT-PCR (qRT-PCR). The microarray data confirmed the specific expression of *B. xylophilus* genes during infection of *P. thunbergii*, which provides basic information that facilitates a better understanding of the molecular mechanism of PWD.

## Introduction

 Pine wilt disease (PWD) is one of the most serious diseases [1,2] that affects coniferous forests around the world and it is considered to be caused by pine wood nematodes (PWNs), *Bursaphelenchus xylophilus* (Steiner & Buhrer) Nickle [3,4]. In Asia, PWD was introduced into Japan during the early 20^th^ century [5] and it spread subsequently to other countries, including China [6] and Korea [7], where pine trees have no natural resistance to *B. xylophilus*. Later, PWD spread to European countries, including Finland [8], Portugal [9], and Russia [10]. *B. xylophilus* is considered to be a native of North America [11], where the local forests are generally resistant or tolerant [12]. *B. xylophilus* causes severe economic, environmental, and social impacts [13,14] in non-indigenous areas and it has attracted attention because of its high pathogenicity in host trees. As the main pathogenic agent of PWD [15], PWNs lead to the death of pine trees within several months, especially between May and September. *B. xylophilus* is vectored by longhorn beetles in the genus *Monochamus* [16–19] and is transmitted via an unusual tree to tree infection route [20]. It is widely known that the introduction and expansion of *B. xylophilus* into non-native areas was mediated mainly by the international wood trade via the short- or long-distance transportation of pine wood, as well as packing materials and wood products infected by PWN [21–23]. Therefore, there is an urgent need to develop effective management strategies to control PWD [24].

 PWN is a serious threat to forests in Asia and Europe, and the risk of this problem is likely to increase due to climate change. However, the pathogenic mechanism of PWD is not clear at present [25]. Little was known about the molecular pathogenicity of *B. xylophilus* until ten years ago, but much progress has been made recently since the development of biotechnology techniques [21]. High-throughput sequencing, which is a powerful method for gene research, has been used to screen resistant genes in trees infected by *B. xylophilus* [26]. Microsatellite markers have also been used to study the genetic diversity of PWN to understand its invasion route and the host colonization process [24]. 

 Information about the pathogenesis-related genes of *B. xylophilus* is essential for understanding the pathogenic mechanism of PWD, but there has been little research in this area. Thus, we compared the differential gene expression of *B. xylophilus* in two growth conditions: growth on *Botrytis cinerea* and after inoculating *Pinus thunbergii* with PWNs. The goal of this study was to analyze the specifically expressed PWN genes involved with the early interactions between *B. xylophilus* and *P. thunbergii*, and to screen the pathogenesis-related genes of *B. xylophilus* using DNA microarrays. Quantitative RT-PCR (qRT-PCR) was used to validate the results obtained using the microarray assays. 

## Materials and Methods

### *B*. *Xylophilus* growth conditions and sampling

*P. thunbergii* seedlings (2 years old) obtained from the greenhouse at Nanjing Forest University were transplanted into pots (30 cm in diameter, 25 cm in height) and maintained with a relative humidity of 70%. The seedlings (height c. 80 cm) were watered every other day and maintained with a photoperiod of 14 h day (25°C) and 10 h night (20°C). The highly virulent AMA3cl strain of PWN was used in the experiment, which was maintained by Lihua Zhu in our laboratory [27]. Two treatments were applied: (i) a suspension of 5000 nematodes (a mixture of adults and juveniles) was used to inoculate the fungus *B. cinerea* (the fungus had been incubated at 25°C for 6 days) on potato dextrose agar medium and grown at 25°C for a further 7 days; (ii) the same amount of nematodes was pipetted into wounds (2 cm in length) in *P. thunbergii* seedlings at about 50 cm above the soil level. The inoculated seedlings were cultivated in the greenhouse for 7 days at 25°C during the daytime and 20°C at night, with 70% humidity. The nematodes were separated from *B. cinerea* and *P. thunbergii* seedlings using a Baermann funnel. Next, *B. xylophilus* was collected by centrifugation at 3000 rpm for 1 min and frozen in liquid nitrogen, before further RNA isolation.

### RNA isolation

The RNA was extracted from frozen nematodes using an RNAprep Kit (Tiangen, China) and purified further with an RNAclean Kit (Tiangen, China), according to the manufacturer’s protocol. The RNA was quantified at 260 nm using a spectrophotometer and examined by electrophoresis on a 1.5% agarose gel.

### Microarray construction, hybridization, and data analysis

The microarray experiments were performed with the help of Shanghai Biotechnology Corporation. In total, 31121 oligonucleotides were synthesized based on the whole genome sequences (obtained from http://www.ncbi.nlm.nih.gov/genome) of *B. xylophilus*. A silane mixture was used to prepare hydrophobic glass slides, which contained exposed hydroxyl groups to facilitate nucleotide combining. The synthesized oligonucleotides were spotted robotically onto the hydrophobic surfaces of the glass slides. The first-strand cDNA was synthesized using a T7-oligo (dT) promoter primer. Subsequently, double-stranded cDNA was produced using a combination of Affinity Script RNase Block Mix, dNTP mix, DTT, and First Strand Buffer. The cRNA was generated from cDNAs using a Genechip IVT Labeling Kit (Affymetrix, USA) and purified with an RNeasy Mini Kit (Qiagen, Germany). The cRNAs were then hybridized at 65°C for 17 h with a microarray that contained 31121 probe sets. After hybridization, the slides were washed with GE wash buffer, according to the manufacturer’s instructions, and scanned using an Agilent microarray scanner (Cat#G2565CA, Agilent Technologies, Santa Clara, CA, USA). The data generated by the scanner were normalized using the Quantile algorithm, Gene Spring software 11.0 (Agilent Technologies, Santa Clara, CA, USA). The gene expression data were analyzed using a *t*-test (*P* < 0.01). Only genes with significantly different expression levels were screened for further analysis. The gene data analysis and functional annotation were performed using SBC Analysis System (http://www.sas.ebioservice.com). The results of microarray analysis were deposited in the NCBI database (Gene Expression Omnibus) and the accession number is GSE50481.

### Quantitative real-time RT-PCR

Real-time quantitative RT-PCR (qRT-PCR) was used to verify the microarray results. Gene-specific primers (Table S1) were designed using Primer Premier 5 software. The actin gene of *B. xylophilus* was selected as the internal control. The first-strand cDNA was synthesized using a Prime Script 1st strand cDNA synthesis Kit (TaKaRa, Japan). The cDNA samples were diluted to 20 ng/µl. Real-time PCR was conducted using a 20 µl reaction volume, which contained 2 µl of template, 10 µl SYBR Premix Ex Tap, 0.4 µl ROX Reference Dye II, 0.4 µl forward primer, 0.4 µl reverse primer, and 6.8 µl ddH_2_O. PCR amplification was carried out using the following conditions: denaturation at 95°C for 30 s, followed by 40 cycles of amplification where each cycle comprised denaturation at 95°C for 5 s, and annealing and extension at 60°C for 34 s. The quantitative variations in the selected genes in the two growth conditions were evaluated using the relative quantification method (ΔΔ CT) [28].

## Results

### Screening of the specifically expressed genes of *B*. *Xylophilus* when cultured on *B. cinerea* and inoculated into *P. thunbergii*

**Figure 1:**
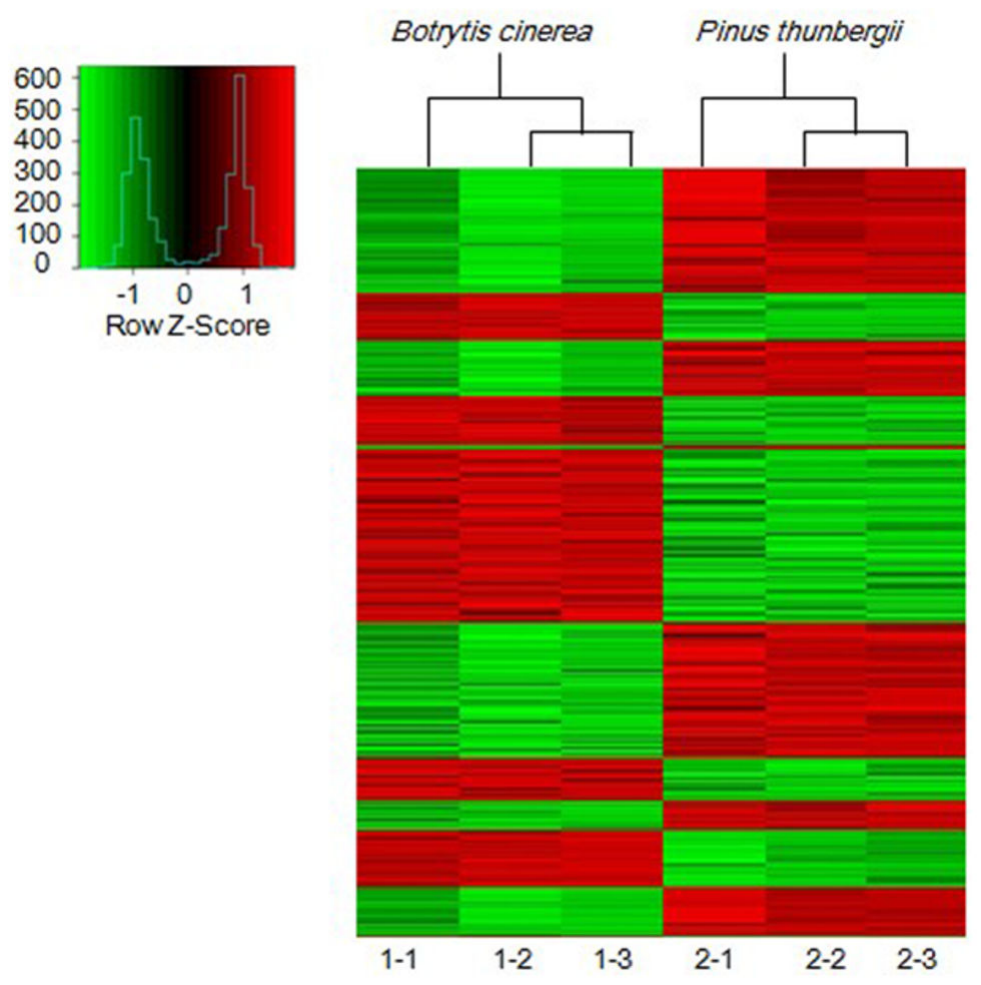
*Bursaphelenchus xylophilus* genes with significantly different expression levels after growth on *Botrytis cinerea* (1-1, 1-2, 1-3) and when used to inoculate *Pinus thunbergii* seedlings (2-1, 2-2, 2-3). The expression data for the 1310 genes that changed significantly were clustered using the SBC Analysis System (http://www.sas.ebioservice.com). The heat map shows the genes that were upregulated (red), downregulated (green), and no change (black).

*B. xylophilus* was separated from *B. cinerea* and *P. thunbergii* seedlings using Baermann funnels. To reduce the likelihood of biological errors, nematodes were collected from five inoculated *P. thunbergii* seedlings that exhibited similar symptoms and combined as one sample. There were three replicates for each treatment. Differences in the transcripts of *B. xylophilus* in the two different growth conditions were detected by genome-wide expression profiling using the Agilent *B. xylophilus* GeneChip microarray containing 31121 probe sets. Over 31000 probe signals were detected from the microarrays and 1310 (4.2%) *B. xylophilus* genes were differentially expressed (Figure 1). Of these 1310 genes, 633 genes were upregulated, whereas 677 genes were downregulated. Of the 633 upregulated genes, 569 genes were upregulated by 2- to 5-fold and 64 genes were upregulated >5-fold. Among the specifically expressed genes, we focused on those related to cell wall degradation, detoxification, and reproduction to identify pathogenesis-related genes.

### Functional annotation

**Figure 2:**
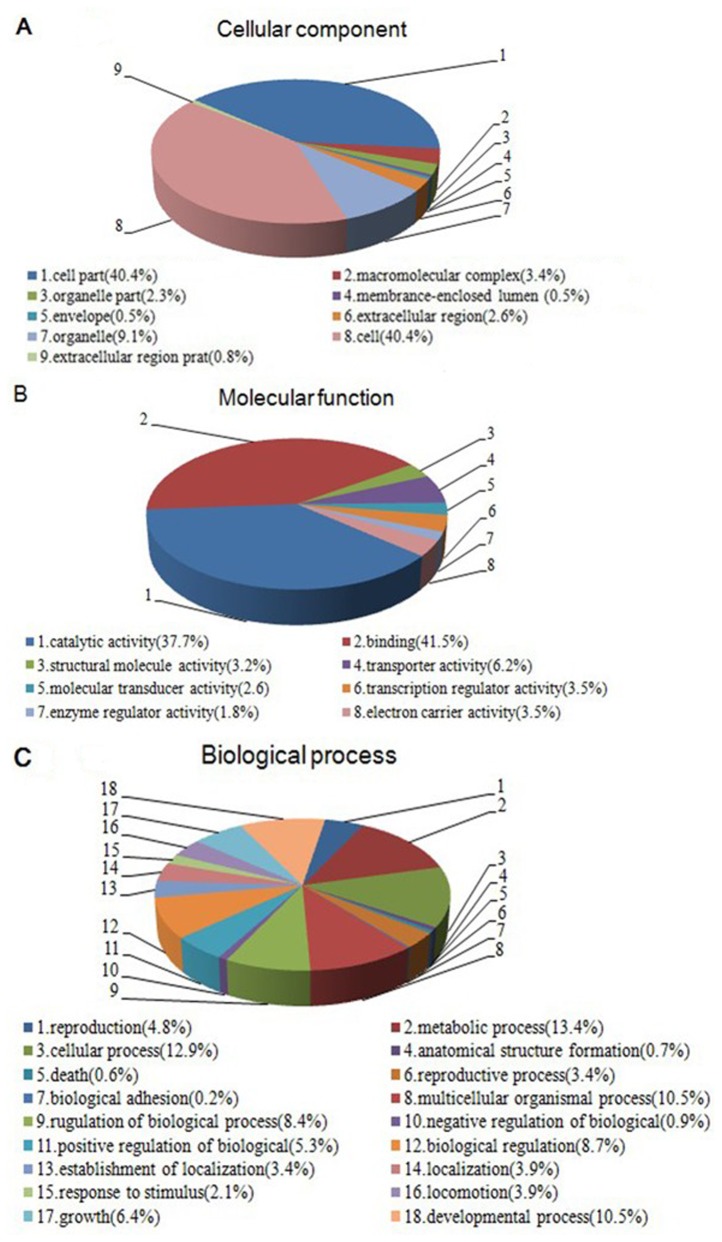
Classification of the annotated genes for *Bursaphelenchus xylophilus*. The genes that changed significantly were divided into three functional sub-categories based on the Cellular Component (A), Molecular Function (B), and Biological Process (C) categories, according to the Gene Ontology (GO) principles.

Functional annotation was performed to assign the genes of *B. xylophilus* with Gene Ontology (GO) terms. The main GO categories included Cellular Component, Molecular Function, and Biological Process. Based on the annotations (Figure 2) for Biological Process, 13.4% and 12.9% of the assignments belonged to the categories “Metabolic Process” (GO: 0008152) and “Cellular Process” (GO: 0009987), respectively, followed by “Developmental Process” (GO: 0032502, 10.5%) and “Multicellular Organismal Process” (GO: 0032501, 10.5%). In addition, the “Binding” (GO: 0005488) and “Catalytic Activity” (GO: 0003824) categories were prominent among the Molecular Function terms with 41.5% and 37.7% of the assignments, respectively, followed by the categories “Transporter Activity” (GO: 0005215, 6.2%), “Transcription Regulator Activity” (GO: 0030528, 3.5%), and “Electron Carrier Activity” (GO: 0009055, 3.5%). Furthermore, the categories of “Cell Part” (GO: 0044464) and “Cell” (GO: 0005623) had the same proportion, followed by “Organelle” (GO: 0043226) and “Macromolecular Complex” (GO: 0032991), which accounted for 9.1% and 3.4% of assignments. Interestingly, a recent study reported a similar classification of the annotated amino acid sequences in *Pinus pinaster* and *Pinus pinea* after inoculation with *B. xylophilus* [28]. In general, the aforementioned GO classes accounted for the majority of the specifically expressed genes. 

**Figure 3:**
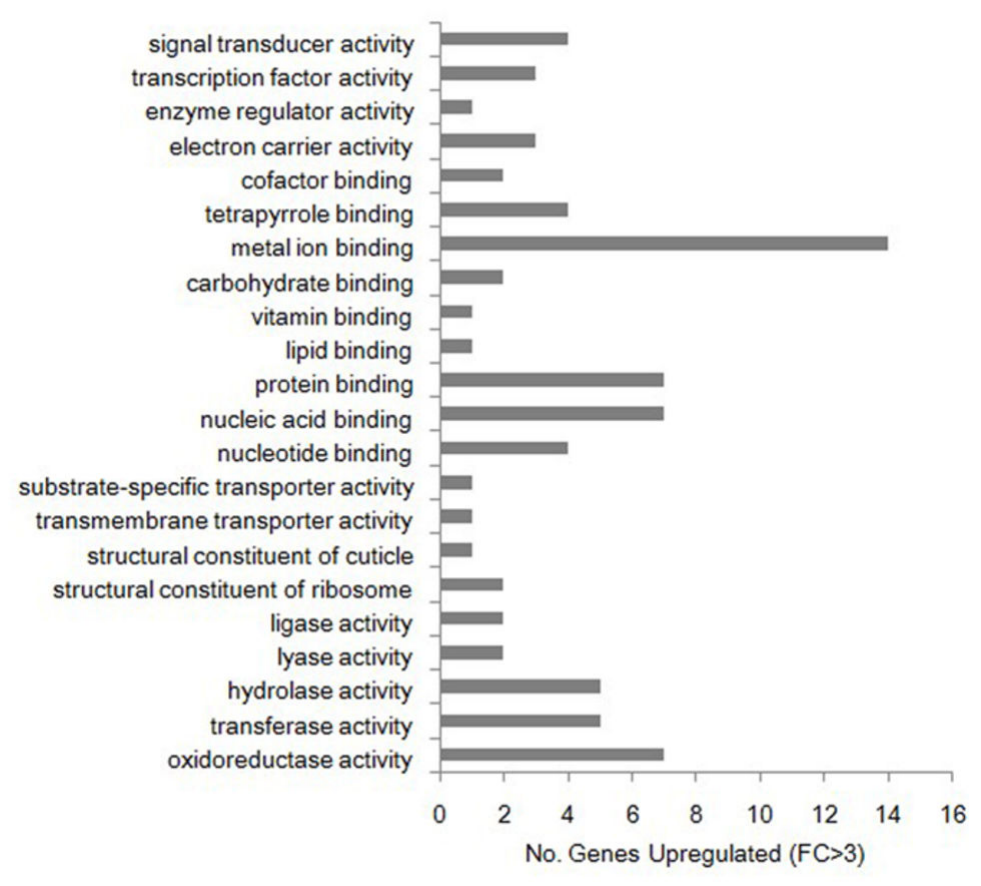
Numbers and Gene Ontology (GO) classifications of genes that changed significantly. The genes that changed significantly were designated as those where the expression level changed by more than 3-fold when *Bursaphelenchus xylophilus* was used to inoculate *Pinus thunbergii* compared with those grown on *Botrytis cinerea*.

The gene expression levels of *B. xylophilus* changed dramatically when the nematode was used to inoculate *P. thunbergii* compared with those grown on *B. cinerea*. We found that 520 genes were upregulated at least 3-fold, including 176 annotated genes. The GO analysis showed that these highly expressed genes (changes of >3-fold) were related mainly to metal ion binding, transferase activity, protein binding, nucleic acid binding, nucleotide binding, oxidoreductase activity, and hydrolase activity (Figure 3). The upregulated metal ion binding-related genes may contribute significantly to the enhancement of various enzyme activities and increase the adaptability of *B. xylophilus*. Interestingly, transferases related to metal ions were also upregulated. The protein binding factors associated with regulation probably activated the over-expression of the pathogenicity-related genes of *B. xylophilus* during the PWD process. The high expression levels of nucleic and nucleotide binding-related genes indicated that *B. xylophilus* can reproduce in *P. thunbergii*, which is the causal premise of PWD. In addition, a number of oxidoreductase and hydrolase genes, such as glucose oxidase, pectate lyase, and glutathione *S*-transferase genes, were detected by the microarrays. According to the functional annotation, we found that some of the cell wall degradation-related genes were upregulated significantly. These genes are considered to be key factors that allow *B. xylophilus* to invade its host. 

### qRT-PCR analysis

**Figure 4:**
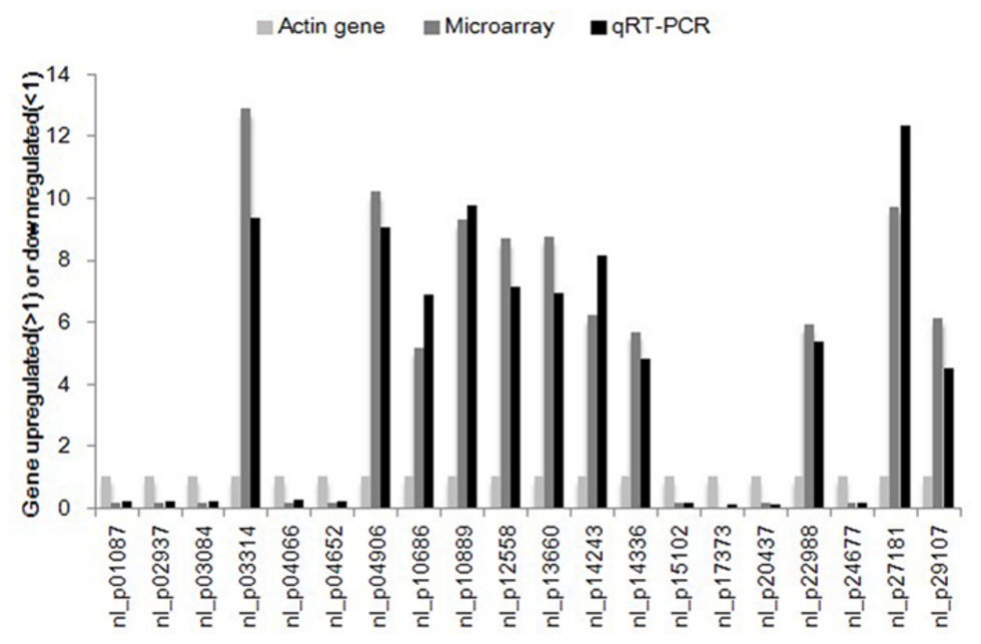
Validation of the DNA microarray results by qRT-PCR. Twenty genes were chosen at random from the highly expressed sequences and their expression levels were assessed by qRT-PCR. The numbers on the horizontal axis represent the probe name in the microarray, while those on the vertical axis are the genes that were upregulated or downregulated.

To validate the microarray results, 20 genes were selected at random from the highly expressed sequences and analyzed by qRT-PCR. We designed specific-primers for nine downregulated genes (Probe name: nl-p01087, nl-p02937, nl-p03084, nl-p04066, nl-p04652, nl-p15102, nl-p17373, nl-p20437, nl-p24677) and 11 upregulated genes (Probe name: nl-p03314, nl-p04906, nl-p10686, nl-p10889, nl-p12558, nl-p13660, nl-p14243, nl-p14336, nl-p22988, nl-p27181, nl-p29107). The expression patterns of the 20 candidate genes detected by qRT-PCR were similar to those detected by the microarray (Figure 4), which demonstrated the reliability of the microarray data.

## Discussion

Valuable information was found by identifying specifically expressed genes based on the differential gene expression of PWNs used to inoculate *P. thunbergii* compared with those grown on *B. cinerea* [26]. The plant cell wall is the primary barrier that a pathogen must penetrate [29]. The cell wall is comprised mainly of pectin, cellulose, and hemicellulose [30]. Cellulose is a polymer of (1–4)-linked β-D-glucose that is interlocked by hemicellulose to form a strong elastic network [31,32]. The network is embedded in pectin, which is the most complex component of the cell wall polysaccharides. The plant cell wall plays a critical role in resisting invasion by *B. xylophilus*. To invade the host successfully, *B. xylophilus* must produce a range of cell wall degradation enzymes that break down this barrier. Therefore, the high level expression of cell wall degradation-related genes is essential to allow *B. xylophilus* to infect its plant host.

 Cell wall-degrading enzymes have been proposed to determine the pathogenicity of PWN. Plant parasitic nematodes have a wide range of cell wall-degrading enzymes, such as cellulase, polygalacturonase [33], pectate lyases [34], and xylanases [35], which contribute to the nematode’s ability to feed on plant tissues. Cellulase is one of the key enzymes associated with the pathogenicity of PWN [36]. Only the GHF5 and GHF45 families of cellulases have been found in nematodes and it has been proposed that they were acquired via horizontal gene transfer (HGT) events [37]. Mayer [38] studied the evolution of the cellulase genes of ten nematode species based on a phylogenetic framework and demonstrated that nematode species with a cellulase gene acquired via HGT exhibited cellulase activities. It has been suggested that the cellulase gene was functionally integrated into the nematode’s genome. In the present study, a number of cellulose degradation-related genes were detected by our microarray analysis, such as beta-1,3-endoglucanase, beta-1,4-endoglucanase, and cellulase (Table 1). The expression of beta-1,4-endoglucanase genes did not change greatly (0.9-fold). However, the expression levels of cellulase genes were downregulated significantly. During the early stage of infection, it is possible that cell wall components other than cellulose are degraded primarily by *B. xylophilus*. 

**Table 1:** Putative cell wall degradation-related genes of *Bursaphelenchus xylophilus*.

**Genes**	**Function**	**Fold change**
cht-1	Chitinase activity	0.1354
BxPel2	Pectate lyase activity	6.6523
Bx-13g-1	Beta-1,3-endoglucanase activity	0.1269
Bx-eng-1	Beta-1,4-endoglucanase activity	0.8897
Bx-eng-2	Beta-1,4-endoglucanase activity	0.8897
Bx-eng-3	Beta-1,4-endoglucanase activity	0.8897
BX-C10	Cellulase activity	0.2666
BX-C12	Cellulase activity	0.2666
BX-C13	Cellulase	0.2666

 Pectin-degrading enzymes, such as polygalacturonases and pectate lyases (PLs), are the primary cell-degrading enzymes secreted by pathogens, which can expose more polymers that need to be degraded by other cell-degrading enzymes [39,40]. PLs are thought to be important pathogenic factors for phytopathogens [41]. A pectate lyase 2（pel-2）gene was detected in *B. xylophilus* based on the microarray assays (Table 1) and its expression was upregulated >6-fold. The high expression of PLs may enhance the infection capacity of *B. xylophilus* and facilitate its migration through plant tissues. Kang et al. (2012) showed that the migration activity of *B. xylophilus* plays an important role in efficient parasitism [42]. The pel-2 gene has also been detected in potato cyst nematodes (PCNs) and experiments demonstrated that the silencing of pel-2 in PCN juveniles reduced the infection efficiency greatly [43]. 

 As one of the primary natural biopolymers, chitin is a major component of fungal cell walls. *B. xylophilus* can secrete chitinase to digest fungal cell walls throughout its growth cycle [44]. The expression of a chitinase gene (cht-1) was downregulated dramatically when *B. xylophilus* was used to inoculate *P. thunbergii* compared with culture on *B. cinerea*, probably because of the change in the available sources that *B. xylophilus* could fed upon. The formation and degradation of chitin are essential for nematode oviposition and molting cycle development. Nematode metamorphosis is regulated by the release of ecdysone, which is related to the regulation of chitin synthesis. A previous report showed that ecdysone plays a role in regulating the expression levels of the CHS-1 and CHS-2 genes during *Drosophila* metamorphosis [45]. However, abnormal chitin synthesis could lead to disorders during nematode development. Several proteins, such as yeast CHS4p and CHS5p, are related to the regulation of chitin synthesis in yeast [46]. Interestingly, the expression of chitinase is restricted to the period of molt and pupation, and different growth conditions [47]. Our microarray results showed that the expression levels of ecdysteroid-related genes were upregulated dramatically when *P. thunbergii* was inoculated with *B. xylophilus*. For example, the ptc-1 and rack-1 genes were upregulated by 4.2-fold and 3.6-fold, respectively. 

 The cytochrome p450 (CYP450) family includes enzymes that play important roles during the biotransformation of secondary metabolites [48]. Studies have shown that *C. elegans* CYP450s catalyze a series of exogenous and endogenous substrates, which cope with variation in the growth conditions [49]. This phenomenon may also occur in *B. xylophilus*. Pine trees generate numerous secondary metabolites, such as terpenoids and cyclic aromatics, to combat the invasion by *B. xylophilus* [26]. *B. xylophilus* must also mobilize defensive reactions to avoid damage by these complex compounds [50,51]. The presence of chemical compounds and plant secondary metabolites in the environment can induce the expression of CYP450 genes [52,53]. Based on complete genome sequencing data, 80 and 76 CYP450 genes were detected in *C. elegans* and *B. xylophilus*, respectively [54]. As shown in Table 2, the expression levels of seven CYP450 genes changed strikingly according to the microarray analysis. Of these, four genes were upregulated by at least 3-fold, whereas three genes were downregulated. It is surprising that the CYP450 genes of *B. xylophilus* not only generate enzymes to utilize secondary metabolites, but they also produce toxic metabolites that damage *P. thunbergii* [32]. Thus, the over-expression of CYP450 genes may enhance the pathogenicity of *B. xylophilus* and play a crucial role in the disease process [55]. However, the pathogenic role of the CYP450 genes in *B. xylophilus* remains unknown [56]. The gene function annotations showed that the W01A11.1 gene of *B. xylophilus*, which was upregulated by 6.3-fold, was a response to toxins, thereby suggesting that *P. thunbergii* generates a range of secondary metabolites as toxins that resist invasion by *B. xylophilus*. Furthermore, the accumulation of secondary metabolites produced by *P. thunbergii* may induce the over-expression of *B. xylophilus* CYP450 genes [57,58].

**Table 2:** Putative detoxification-related genes of *Bursaphelenchus xylophilus*.

**Genes**	**Function**	**Fold change**
ugt-49	UDP-Glucuronosyl Transferase	3.1834
ugt-47	UDP-Glucuronosyl Transferase	3.5447
ugt-59	UDP-Glucuronosyl Transferase	3.3254
ugt-54	UDP-Glucuronosyl Transferase	7.509
cyp-31A3	Monooxygenase activity	0.3014
cyp-25A5	Monooxygenase activity	0.1778
cyp-13A11	Monooxygenase activity	3.0041
cyp-33C1	Monooxygenase activity	0.3102
cyp-33C4	Monooxygenase activity	4.4049
cyp-33C9	Monooxygenase activity	6.2141
cyp-33D3	Monooxygenase activity	3.2066
abtm-1	ABC Transporter	3.8127
gst-33	Glutathione S-Transferase	3.3241
W01A11.1	response to toxin	6.3557
Y52B11A.8	lipid catabolic process	5.1726

 At present, the detoxification process of *B. xylophilus* is divided into three phases [54]. First, CYPs are the primary proteins in the first phase, which provide the enzyme substrates for the next stage. Second, the glutathione S-transferases (GSTs) and UDP-glucuronosyl transferases (UGTs) are essential in the second phase. A series of detoxification reactions occurs during this stage, which produce a high level of efflux. As shown in Table 2, one GST and four UGTs were found to be upregulated by at least 3-fold. In the last phase, ATP-binding cassette (ABC) transporters are the main group responsible for the efflux of detoxified molecules. One ABC transporter was found to be highly upregulated according to the microarray analysis.

 In particular, the expression levels of genes related to detoxification were upregulated significantly, which suggests that *B. xylophilus* enhanced its gene expression in response to the secondary metabolites produced by pine trees. One gene, Y52B11A.8, which is related to the lipid catabolism process, was detected by the microarray. The expression of Y52B11A.8 was upregulated by more than 5-fold. This indicated that lipids, which are likely to have nematocidal activity, were produced by pine trees after invasion by *B. xylophilus*.

 The reproductive ability of *B. xylophilus* in pine trees is a crucial causal factor in PWD [56]. Many reports have suggested that the virulence level of *B. xylophilus* is associated with its reproductive ability in *in vitro* or in vivo conditions [59,60]. When two PWN isolates (virulent and avirulent) were inoculated into pine trees, the PWN population of the virulent isolate was greater than that of the avirulent isolate. The rate of population increase by the virulent isolate was also greater than that of the avirulent isolate in PWD-killed seedlings [60–62]. The translocation of the virulent isolate into the xylem resin canals and phloem tissues was faster than that of the avirulent isolate after they were inoculated into separate pine trees [63]. Aikawa et al. [64] reported that the number of third- and fourth-stage dispersal juveniles (JIIIs and JIVs) of a virulent isolate from adult beetles was much higher than that of the avirulent isolates. This indicated that the fertility of the virulent isolate was higher than that of the avirulent isolate. Previous studies have shown that the virulence of nematodes is closely correlated with their reproductive ability [65]. The reproductive ability of *B. xylophilus* in host trees may be an important marker of its virulence, which could be used to evaluate the virulence level of *B. xylophilus*. A series of reproduction-related genes were detected by the microarray analysis (Table 3). The functions of reproduction-related genes were involved with egg hatching, germ cell development and meiosis, and the positive regulation of the growth rate, etc. The results indicated that the expression levels of the reproduction-related genes of *B. xylophilus*, such as rpl-24.2, R11A8.2, set-16, and F25H5.6, were upregulated dramatically by growth in *P. thunbergii* compared with those when grown on *B. cinerea*. 

**Table 3:** Putative reproduction-related genes of *Bursaphelenchus xylophilus*.

**Genes**	**Function**	**Fold change**
vps-34	nematode larval development	4.0836
ngp-1	genitalia development	3.5102
F25H5.6	morphogenesis of an epithelium	4.3557
rpl-24.2	body morphogenesis	8.386
vps-32.1	embryonic development ending in birth or egg hatching	3.3897
ptc-1	oviposition and molting cycle	4.2054
rsr-2	nematode larval development	4.0309
rpl-16	structural constituent of ribosome	3.3006
set-16	nematode larval development	5.4682
hoe-1	germ cell development and meiosis	3.1255
rack-1	oviposition and genitalia development	3.6128
R11A8.2	positive regulation of growth rate	6.194
his-66	nucleosome assembly	3.6435
mua-1	nematode larval development	4.0318

 In the early stages of PWD, ligno-suberization and wound periderm were observed around the cortex resin canal. In the advanced stage, the number of nematodes increased dramatically and the cambium of pine trees was destroyed [25,66]. The results of previous studies have shown that *B. xylophilus* feeds mainly on the xylem ray parenchyma cells of pine trees [67]. The rapid reproduction rate of *B. xylophilus* in *P. thunbergii* probably leads to more serious destruction of the cortex resin canal. However, more of the cortex parenchyma was fed on by *B. xylophilus*, which induces rapid increases in the cavitation area. The accumulation of the toxic substances produced by *B. xylophilus* also aggravated the wilting of needles. Thus, a high frequency of nematode reproduction is probably an important pathogenicity factor related to PWD. 

 PWD is a complex disease and its mechanism remains unclear. The specific genes related to cellulose degradation, hydrolase, and detoxification by *B. xylophilus* may play key roles in this disease. Thus, the mechanism of PWD may be involved with variation in the transcript levels of *B. xylophilus* genes. Our microarray analysis demonstrated that the expression levels of *B. xylophilus* genes changed significantly after it was used to inoculate *P. thunbergii* seedlings. 

 The present study investigated the differential gene expression of *B. xylophilus* cultured on *B. cinerea* and when used to inoculate *P. thunbergii*. The results showed that the gene expression of *B. xylophilus* changed significantly and a number of genes were upregulated dramatically. We found that some of the upregulated genes were related to cell wall degradation and reproduction, which suggests that *B. xylophilus* not only succeeded in invading *P. thunbergii*, but it also reproduced within *P. thunbergii*. In addition, we detected several toxin-related genes, which indicate that *B. xylophilus* may produce toxins and utilize the secondary metabolites of its host, and this process is possibly involved with the wilting of pine trees. These data facilitate a better understanding of the molecular mechanism of PWD and they may help to develop effective control strategies to combat *B. xylophilus*. However, further investigations are required to understand the functions of these specifically expressed genes.

## Supporting Information

Table S1 **Primers used in the quantitative real-time PCR (qRT-PCR) analysis.**
(DOCX)
