# Supplementary material for: Specifically Expressed Genes of the Nematode Bursaphelenchus Xylophilus Involved with Early Interactions with Pine Trees
Source: PLoS One. 2013 Oct 14;8(10):e78063. doi: 10.1371/journal.pone.0078063 (PMC3796492; doi:10.1371/journal.pone.0078063)
Supplement: Table S1 — Primers used in the quantitative real-time PCR (qRT-PCR) analysis. (DOCX) [file pone.0078063.s001.docx]

**Supporting Information**

**Table S1.** Primers used in the quantitative real-time PCR (qRT-PCR) analysis

|  |  | |  |
| --- | --- | --- | --- |
| **Probe name** | | **Sense** | **Anti-sense** |
| Actin | 5’-GCAACACGGAGTTCGTTGTA-3’ | | 5’-GTATCGTCACCAACTGGGAT-3’ |
| nl_p01087 | 5’-AGTTCAATTCTACTACGGTTG-3’ | | 5’-CTTTGTAAGGTTTCGAGTTCT-3’ |
| nl_p02937 | 5’-AAACGAAACTCCCAGTCCAG-3’ | | 5’-CCTGATTGTTACCTCCTCCC-3’ |
| nl_p03084 | 5’-CCCAGCAAACAGTCAGAACA-3’ | | 5’-AATTTACAACCGCGAAATCA-3’ |
| nl_p03314 | 5’-TCCGTCATCACTAATTCACC-3’ | | 5’-ATGGCAAGTTTCAGCATTTT-3’ |
| nl_p04066 | 5’-AGATGTAGATACCAATGGGA-3’ | | 5’-GAGAAGAATTTGAGGAGGAA-3’ |
| nl_p04652 | 5’-CAATGGACCTGATGGAGAAC-3’ | | 5’-ATGAAGGCGATAAGAAATGG-3’ |
| nl_p04906 | 5’-CTGAAACTTGCCGTGCTAAA-3’ | | 5’-GACGGAGTGAATTGACAAGA-3’ |
| nl_p10686 | 5’-TCGATCAAGCCCACGACTGT-3’ | | 5’-CGGTTCATCAGCGGGACATA-3’ |
| nl_p10889 | 5’-ATGATGTATGCTTAGGGAGGG-3’ | | 5’-CACAATGGCGATAGAAATGG-3’ |
| nl_p12558 | 5’-TCCGTCATCACTAATTCACC-3’ | | 5’-AATCAAACTACGCCGACACT-3’ |
| nl_p13660 | 5’-GCTGAAACTTGCCACGCTAA-3’ | | 5’-GACGGAGTGAATTGACAAGA-3’ |
| nl_p14243 | 5’-ATGTGGGTGGCACCAAGTCT-3’ | | 5’-GCGGCATCTACAACGACCAT-3’ |
| nl_p14336 | 5’-CCAGTCGTGGACAACAAAGT-3’ | | 5’-AGTCCGACGCAAGCGAGGGT-3’ |
| nl_p15102 | 5’-GCGAAGAAGAATGGGTTTGA-3’ | | 5’-CCTTGATGAGTTGCGTGAAT-3’ |
| nl_p17373 | 5’-ATGGGTTCTGTCAGCTTCTTT-3’ | | 5’-AATGTCGGTGTTGTCTTGGA-3’ |
| nl_p20437 | 5’-AAAGTGGTCGGCTTTGCTAT-3’ | | 5’-TTTGGGAAGTCGGATTCATT-3’ |
| nl_p22988 | 5’-CTTATCACCCAGGCCCATCA-3’ | | 5’-CTTCAGCAGCCAATTCAGCA-3’ |
| nl_p24677 | 5’-GATTTGGGAGGACGACTTCA-3’ | | 5’-GCTGTCCGTATAGCATTGGAG-3’ |
| nl_p27181 | 5’-CCAAACTGGACGGCACAACT-3’ | | 5’-GCCCACGGACTCGAAGAAAG-3’ |
| nl_p29107 | 5’-TATCGCATTGATGAAGAGGGT-3’ | | 5’-AGCCGTTGGTGATGAGACAT-3’ |
